# Soil amendment and rhizobacterial inoculation improved Cu phytostabilization, plant growth and microbial activity in a bench-scale experiment

**DOI:** 10.3389/fmicb.2023.1184070

**Published:** 2023-06-29

**Authors:** Marc Romero-Estonllo, Judith Ramos-Castro, Yaiza San Miguel del Río, Beatriz Rodríguez-Garrido, Ángeles Prieto-Fernández, Petra S. Kidd, Carmen Monterroso

**Affiliations:** ^1^CRETUS, Departamento de Edafoloxía e Química Agrícola, Universidade de Santiago de Compostela, Santiago de Compostela, Spain; ^2^Misión Biológica de Galicia Sede de Santiago de Compostela (MBG), Consejo Superior de Investigaciones Científicas (CSIC), Santiago de Compostela, Spain

**Keywords:** mine tailings, phytoremediation, soil amendment, *Populus nigra*, co-cropping, PGPR, mycorrhiza, soil microbial community

## Abstract

Mine driven trace elements’ pollution entails environmental risks and causes soil infertility. In the last decades, *in situ* techniques such as phytostabilization have become increasingly important as ways to tackle these negative impacts. The aim of this study was to test the individual and combined effects of different aided phytostabilization techniques using substrate from barren tailings of a Cu mine, characterized by extreme infertility (high acidity and deficiency of organic matter and nutrients). The experiment analyzed the growth of *Populus nigra* L. planted alone (P) or in co-cropping with *Trifolium repens* L. (PT), in pots containing mine soil amended with compost (1, 10, compost, soil, w/w) non inoculated (NI) or inoculated with plant growth promoting rhizobacteria (PGP), mycorrhizae (MYC) or a combination of bacterial and fungal inocula (PGPMYC). Non-amended, non-planted and non-inoculated reference ports were also prepared. Plants were harvested after 110 days of plant development and several biometric and phytopathological parameters (stem height, aerial biomass, root biomass, wilting, chlorosis, pest and death) and macro and micronutrient composition were determined. The growth substrate was analyzed for several physicochemical (pH, CEC_e_, and exchangeable cations, total C and N, P Olsen and availability of trace elements) and microbiological (community level physiological profiles: activity, richness and diversity) parameters. The use of the amendment, *P. nigra* plantation, and inoculation with rhizobacteria were the best techniques to reduce toxicity and improve soil fertility, as well as to increase the plant survival and growth. Soil bacterial functional diversity was markedly influenced by the presence of plants and the inoculation with bacteria, which suggests that the presence of plant regulated the configuration of a microbial community in which the inoculated bacteria thrive comparatively better. The results of this study support the use of organic amendments, tolerant plants, and plant growth promoting rhizobacteria to reduce environmental risk and improve fertility of soils impacted by mining.

## 1. Introduction

In mine dumps, high concentrations of trace elements, acidity, scarcity of organic matter, low nutrient availability, poor water holding capacity and substrate structure frequently hinder plant colonization and establishment (Epelde et al., 2014; Wang et al., 2017). These areas are unproductive and hazardous as potential sources of contamination, so their remediation serves a dual purpose: to recover these lands for productive use and to ensure environmental safety (Ali et al., 2013; Ortiz-Hernández et al., 2018). Trace elements (TEs), which include heavy metals and metalloids, are usually scarce in the lithosphere, and constitute important inorganic pollutants in mine areas (Lago-Vila et al., 2014; Ortiz-Hernández et al., 2018). Some TEs are micronutrients, but at relatively high concentrations they become toxic, affecting a multitude of physiological functions (Kabata-Pendias and Pendias, 2001; Martínez-Martínez et al., 2019).

Traditional remediation techniques, that are based on excavation of contaminated soil and off-site (*ex situ*) treatment, are expensive both in materials and labor and cause irreversible alteration to the soil physic-chemical and microbiological properties (Ali et al., 2013). In recent decades, techniques for remediation of contaminated soils have diversified and the so-called Gentle soil Remediation Options (GROs) have been developing as attractive alternatives from economic, environmental and social points of view (Ali et al., 2013; Kidd et al., 2015; Sarwar et al., 2017). Among these techniques, aided phytoremediation is a GRO based on the combined use of plants and their associated microorganisms, aided through the use of edaphic amendments and other agronomic practices (co-cropping, bioaugmentation, watering, pesticides, etc.). In the case of mine soils, the use of metal(loid)-tolerant plants and the incorporation of organic and inorganic amendments to facilitate plant establishment, and to directly reduce metal mobility and availability, are key for the success of the strategy adopted, frequently phytostabilization treatment (Kumpiene et al., 2014; Clemente et al., 2015). The development of vegetation cover in mine tailings is essential for protecting the soil from erosion and decreasing the mobility of pollutants and mitigating the impact of pollution on the adjacent ecosystems (Arranz-González, 2019). On the other hand, the constant injection of photosynthates from established plant cover in the soil helps to counteract carbon mineralization of amendments over time (Asad et al., 2019). The phyto-based techniques of soil remediation allow the *in situ* inactivation of pollutants preventing further spread and transfer to the food chain (Vangronsveld et al., 2009; Touceda-González et al., 2017), while restoring biogeochemical components and processes that are the foundation of the services provided by the ecosystem (i.e., carbon sequestration, water quality, renewable biomass, landscape amenity and others; Becerra-Castro et al., 2018).

The proper selection of plant species and cultivation systems is very important for the success of phytostabilization. Plants must: thrive in the climatic conditions of the geographical area of intervention, have the ability to grow in poor soils with nutrient limitation or adverse pH, tolerate high levels of contaminants, avoid accumulating pollutants in the aerial part and possess a dense and deep root system to physically support the soil. It is desirable that they also have rapid growth and high biomass production. The use of local plants is advisable because they are already adapted to the conditions of the place, as well as to pests and other stressors (Gerhardt et al., 2017). Several species in the *Salix* and *Populus* genera are considered good candidates for phytostabilization techniques due to their deep root system, tolerance to metals and nutrient deficiency, and high resprouting capacity from stumps after harvests (Kidd et al., 2015) which allows their establishment as dense, short rotation coppices (SRC) covering almost the entire soil surface and yielding a large amount of biomass (Štochlová et al., 2019). The harvested biomass can be used for bioenergy, lumber production or biofortified products, etc., (Conesa et al., 2012). The SRC have also limitations such as high water demand and enhanced phytopathogen and pest susceptibility (Kidd et al., 2015; Yáñez et al., 2019).

Crop diversity mitigates some of the risks associated with monoculture. Mixed cropping patterns using legumes are particularly interesting because many species have the ability to fix atmospheric N_2_ through symbiotic bacteria (De Antoni Migliorati et al., 2015). Legumes are widely used in agriculture and their incorporation in the remediation process can improve the growth and nutritional status of metal-tolerant plant species (Kidd et al., 2015; Arranz-González, 2019).

Soil organic amendments such as municipal sludge, residues or by-products of industrial and agri-food processes, provide organic matter and nutrients, contribute to correct extreme pH, improve physical properties (reducing compaction and increasing water holding capacity) and promote microbial activity (Becerra-Castro et al., 2018). In addition, amendments can modify the bioavailability of metal(loid)s in soil through adsorption, complexation, chelation, precipitation, reduction and volatilization (Park et al., 2011; Clemente et al., 2019).

In the case of mine tailings and other metal enriched substrates the influence of microorganisms on the bioavailability of metals in the soil by modifications in the conditions of solubility in the medium, excretion of chelators, binding to wall surface molecules, etc. is also of great importance (Sessitsch et al., 2013; Ortiz-Hernández et al., 2018). Additional microbial benefits can be obtained inoculating selected rhizobacterial strains with plant growth promoting properties (PGPR; e.g., phosphorous solubilization, nitrogen fixation, production of phytohormones, stimulation of plant immune system, niche competition with plant pathogens, etc.; Nehl et al., 1997; Glick, 2014; Becerra-Castro et al., 2018; Etesami and Maheshwari, 2018) or symbiont fungi such as mycorrhizal fungi able to improve plant nutrition by facilitating nutrient uptake by roots, influencing the production of phytohormones, acting against pathogens or participation in the formation of soil aggregates (Rillig and Mummey, 2006; Marschner, 2011). From the phytostabilization point of view bioinoculation with bacteria and mycorrhizae is regarded as a tool to improve plant growth without the need of impractical or expensive external inputs (Kidd et al., 2015; Asad et al., 2019; Basu et al., 2021).

The objective of this work is to investigate different approaches for improving phytotechnologies useful for the remediation of Cu mine tailings. The experiments were designed to analyze two planting patterns (poplar grown in monoculture or in co-cropping with clover) and the potential benefits of the application of bioinocula (a plant growth promoting rhizobacterial consortium and a mycorrhizal commercial preparation applied separately or in combination).

## 2. Materials and methods

### 2.1. Experimental set-up

#### 2.1.1. Substrate and inocula preparation

The degraded soil used in this study was collected in a mine tailing deposit of a former copper mine. The mine, located in Touro-O Pino (A Coruña (NW Spain); 42°52′34”N, 8°20′40”W), operated over a deposit of shales and amphibolites enriched in metallic sulfides from 1972 to 1986. Previous studies found out that metallic sulfides oxidation caused extremely acidic soils and waters (pH = 2–3), with high electric conductivity and concentration of Fe, Al and trace elements, which completely prevent the growth of vegetation unless soil amendments are applied (Touceda-González et al., 2017). The amendment selected for this study was compost prepared from sewage sludge and food waste material by the company Tratamientos Ecológicos del Noroeste S.L.

To prepare the substrate for the pot experiments the collected mine soil was air-dried, sieved at 8 mm, and homogenized. In parallel, the compost was air-dried and homogenized. Next, over 100 kg of mine soil material were mixed with compost at a ratio of 1:10 (compost:soil; w/w). Aliquots of 1 kg of both non-amended and compost-amended soil were sieved at 2 mm for general characterization. The amended substrate was then wetted to 80% of its water holding capacity and allowed to stabilize at 25°C for 15 days. In the same way, about 10 kg of non-amended soil were also wetted and allowed to stabilize to be used as reference.

After the stabilization period, the amended soil was distributed in 60 pots of 1.5 kg and the non-amended reference substrate was used to prepare 4 reference pots.

Bacterial strains were isolated and characterized by the Soil Microbiology Group of the “Misión Biológica de Galicia” (MBG) of the Spanish Council of Scientific Research (CSIC) from a mine tailing and a TE contaminated soil (Becerra-Castro et al., 2012; Kidd et al., 2021). The selected bacterial strains presented phenotypes involved in the promotion of plant growth of *Populus* spp., namely, *Bacillus* sp. SK2.3 (IAA producer), *Rhodococcus* sp. SK12.6 (IAA and biosurfactant producer, and inorganic phosphate solubilizer), *Streptomyces* sp. SK20.12 (IAA and siderophore producer), and *Massilia niastensis* P87 (IAA producer).

For inoculum preparation, the four bacterial strains were grown separately in 869 medium (containing per liter tryptone, 10 g, yeast extract, 5 g, NaCl, 5 g, D-glucose, 1 g, CaCl_2_-2H_2_O, 0, 35 g) at 28°C on an orbital shaker with 150 rpm agitation until early stationary phase. The bacterial cultures were centrifuged at 5000 g for 10 min (AvantiTM J-30I Centrifuge model), washed twice with 10 mM MgSO_4_ and re-suspended in 10 mM MgSO_4_, adjusting optical density at 590 nm to 0.8–1.0 (model CO7500 colorimeter WPA). Previously, the colony forming units (CFUs) of each bacterial culture was quantified by plate culturing 1/10 serial dilutions. A total of 1 L of inoculum was prepared with equal amounts of each strain.

The mycorrhizal inoculum consisted of a commercial mixture between endo- and extomycorrhizal fungi supplied by the company INOQ GmbH, Germany.

#### 2.1.2. Inoculation, planting, and harvest

The study carried out tested 2 plantation patterns: poplar (*Populus nigra* L.) in a monoculture (P) or in co-cropping with clover (*Trifolium repens* L.) (PT); and 3 inoculation treatments: (i) the combination of plant growth promoting rhizobacteria strains (PGP) described above, (ii) a commercial mycorrhiza (MYC), and (iii) a combination of both inoculants (PGPMYC). Non-inoculated (NI) P and PT and unplanted (NP) pots non-inoculated or receiving the different inocula were also prepared. The experimental design included 5 replicates of each of the treatments assayed, as well as 4 additional unplanted pots containing non-amended, non-inoculated mine soil.

The PGP inoculum was applied by immersing the base of *P. nigra* clonal cuttings (one per pot, about 20 cm long) in the bacterial suspension described above, for 24 h. Cuttings used for the NI and MYC treatments were immersed in 10 mM MgSO_4_. The application of PGP inoculum was repeated after 28 days of plant growth in the glasshouse, by adding 50 mL of bacterial suspension at the base of each *P. nigra* plant. At this stage, plants of the NI and MYC treatments received 50 mL of 10 mM MgSO_4_.The commercial mycorrhizal inoculum of the treatments MYC and PGPMYC was added around the cuttings at the same time of planting following the recommendations given by the supplier (20 mL/pot).

Commercial seeds (about 5 g) of *T. repens* were directly sown at the time of planting poplar cuttings in non-inoculated and inoculated PT pots.

The pots were distributed in random blocks in the glasshouse and were watered every week up to field capacity. After a growth period of 110 days the plants were harvested carefully separating the soil and the plant tissues. After a careful homogenization, an aliquot of soil from each pot was air dried and sieved at 2 mm for physic-chemical analysis. The remaining soil was sieved at 4 mm and stored at 4°C for microbiological determinations. Plant material was treated and analyzed as described later on.

### 2.2. Physic-chemical analyses on soil

General physic-chemical properties and metal availability were analyzed in the non-amended and amended soil materials before and after the planting and inoculation using the air dried fraction <2 mm. Soil pH in distilled water and 0.1 N KCl (solid:solution ratio, 1:2.5) was measured with a METROHM 632 pH meter. Available phosphorus was quantified after extraction with 0.5 M NaHCO_3_, pH 8.2 (Olsen, 1954) and subsequent determination by colorimetry of the phosphomolybdic blue complex (Murphy and Riley, 1962) using a Biochrom Libra S60 spectrophotometer. Exchangeable cations were extracted with unbuffered 1 M NH_4_Cl (Peech et al., 1974) and determined by inductively coupled plasma optical emission spectrometry (ICP-OES, model Vista-PRO). The effective cation exchange capacity (CEC_e_) was calculated as the sum of the exchangeable Ca, Mg, K, Na and Al. The bioavailable content of K, Ca, Mg, Co, Fe, Mn, Zn, Cu, Ni, Pb, Cr and Cd was measured in 1 M NH_4_NO_3_ and 0.05 M EDTA (pH 4.6) extracts (ratio 1:2.5, soil:solution, 2 h agitation) by ICP-OES. Finally, C and N contents were determined by combustion of the ground sample encapsulated in tin foil in a LECO CHN200 analyzer. In addition, the pseudo-total content of potentially toxic metals (Mn, Ni, Cr, Co, Cu, Zn and Pb) was determined by ICP-OES in the non-amended soil used for the experiments after acid digestion with “aqua regia” (HNO_3_ + HCl) in a microwave oven (MILESTONE, model ETHOS, Italy).

### 2.3. Study of the microbial community

The functional diversity of the soil bacterial communities (community level physiological profiles CLPP) was analyzed with BIOLOG EcoPlates™ as described by Pardo et al. (2018). Briefly, fresh soil (sieved at <4 mm and stored at 5° C) suspensions were preparated with sterile 1% sodium hexametaphosphate (HMP) at 1:10 ratio (w/v) and agitation in a rotating shaker (125 rpm) for 30 min. These initial suspensions were 1:10 (w/v) serially diluted with 1% phosphate buffer saline (PBS, pH 7.4) and the 10^−2^ dilution was used to inoculate the plates (125 μL/well). The plates were incubated at 26°C for 1 week and the color development was recorded every 24 h with a microplate reader (PowerWave XS2, BioTek Instruments, United States) at 595 nm.

The average color development per well (AWCD) was determined as the mean of the absorbance value of the wells corrected with the values read at time zero and the values read in the water blank wells (Shannon, 1948; Margalef, 1958; McInthosh, 1967; Grayston et al., 2004). Metabolic richness (S) was considered the number of wells with absorbance >0.15. Margalef index of richness [M = (S − 1)/(logN)], Shannon-Wiener index of diversity (H = −Σp_i_log2p_i_) and McIntosh index of dominance (I = (N − √Σn_i_^2^)/(N − √N)) were calculated after 168 h of incubation considering the absorbance as the number of individuals (n_i_) of each metabolic species and the sum of absorbances as the total number of individuals (N = Σn_i_).

### 2.4. Analyses of plant growth and ionome

The height of the harvested poplar plants was measured and phytopathological symptoms of each specimen were recorded. The plants were then separated into root and shoots. The plant material was washed, dried at 40°C and weighed. Finally, the leaves (with petiole) were also separated and weighed, and the roots, leaves and stems were ground and analyzed separately.

Plant material (about 500 mg) was digested in acid (HNO_3_ 65%: ratio 20:1) in a PerkinElmer® SPB 50–48 block digester, with progressive heating up to 90°C. The concentration of P, K, Ca, Mg, Co, Fe, Mn, Zn, Cu, Ni, Pb and Cd was determined in the digestate obtained by inductively coupled plasma optical emission spectrometry (ICP-OES, model Vista-PRO). Total carbon and nitrogen were determined by combustion of the ground samples in a LECO CHN-200.

### 2.5. Statistical analysis

Multiple one-way analyses of variance (1-ANOVA) and two-way comparison of the means (Tukey test) were applied to analyze the statistical significance of the differences among all the individual treatments. The confidence level of significance considered was 5% (α = 0.05) unless otherwise specified. Homoscedasticity and normality were previously checked with the Levene test and the Holm’s adjusted Shapiro–Wilk test, respectively. Multiple two-way analyses of variance (2-ANOVA) were applied to determine the percentage of variance explained by either the cropping pattern (factor “plant”), the inoculation (factor “inoculum”) or the interaction (“inoculum-plant”). All calculations were performed in “R Commander”, a graphical user interface (GUI) for the free and open-source statistical software “R v. 4.3.0” (R Core Team, 2023). The bacterial carbon substrate degradation patterns were grouped according to a Spearman correlation using the “heatmap.2” function of the R package “gplots”.

## 3. Results

### 3.1. Physic-chemical properties of non-amended and compost-amended soil

In accordance with the geochemical characteristics of the area and the mining activities, the mine soil material used in this study was extremely acidic, with a low effective cation exchange capacity (CEC_e_), and the exchange complex dominated by H^+^ and Al^3+^ and a low saturation in basic cations (< 10%). In addition, these materials were poor in C, N and available P, and rich in total and available Fe, Al and Cu. Due to its observed low fertility this material is hereafter referred to as barren soil (Table 1). As expected, the characteristics of the barren soil did significantly not change in the pots maintained in the glasshouse during the experiment.

**Table 1:** General physic-chemical parameters of barren soil materials, the mixture with compost and the compost itself (mean values and standard deviation).

	Barren soil		Amended soil		Compost	
	Mean	±S.D.	Mean	±S.D.	Mean	±S.D.
pH_H2O_	3.4	0.0	4.3	0.0	6.2	0.0
pH_KCl_	3.3	0.0	4.3	0.0	6.2	0.0
C (%)	1.03	0.03	3.22	0.03	15.40	0.40
N (%)	0.07	0.00	0.33	0.01	1.40	0.01
C/N	14.22	0.65	9.67	0.20	11	–
P_Olsen_ (mg/kg)	10.46	0.41	42.24	1.27	425.30	33.00
CEC_e_ (cmol^+^/kg)	7.01	0.19	8.61	0.61	57.60	4.50
H^+^	4.61	0.71	–	–	–	–
Al^+3^	6.06	0.16	0.92	0.10	–	–
Ca^+2^	0.46	0.03	4.77	0.46	40.40	1.20
K^+^	0.12	0.01	0.59	0.05	4.40	0.10
Mg^+2^	0.33	0.01	2.15	0.18	10.30	0.50
Na^+^	0.04	0.00	0.17	0.01	2.50	0.10
**Pseudo-total concentration of elements (acid digested)**						
Al_T_ (g/kg)	39.3	1.7	35.3	0.1	31.5	0.4
Fe_T_ (g/kg)	117.9	1.8	113.7	0.3	40.0	7.0
Mn_T_ (mg/kg)	748.3	31.4	793.7	3.2	861.0	20.0
Cu_T_ (mg/kg)	391.7	4.2	375.3	1.7	529.0	84.0
Zn_T_ (mg/kg)	131.0	0.6	145.3	0.6	530.0	17.0
Pb_T_ (mg/kg)	13.7	0.4	16.5	1.4	–	–
**Potentially available concentration of elements (EDTA extractable)**						
Al_EDTA_ (mg/kg)	530.45	9.07	371.59	10.44	–	–
Fe_EDTA_ (mg/kg)	387.13	3.30	505.56	8.50	–	–
Mn_EDTA_ (mg/kg)	15.55	2.56	28.45	2.35	–	–
Cu_EDTA_ (mg/kg)	42.31	0.49	37.56	0.66	–	–
Zn_EDTA_ (mg/kg)	2.15	0.24	37.85	2.09	–	–
Pb_EDTA_ (mg/kg)	0.28	0.05	2.42	0.05	–	–
**Readily available concentration of elements (NH**_**4**_ **NO**_**3**_ **extractable)**						
Al_NH4NO3_ (mg/kg)	462.35	4.23	57.37	0.61	–	–
Fe_NH4NO3_ (mg/kg)	1.99	0.06	2.85	0.23	–	–
Mn_NH4NO3_ (mg/kg)	12.18	0.27	17.09	1.21	–	–
Cu_NH4NO3_ (mg/kg)	21.56	0.27	2.45	0.01	–	–
Zn_NH4NO3_ (mg/kg)	1.84	0.02	11.88	0.27	–	–
Pb_NH4NO3_ (mg/kg)	0.04	0.01	0.01	0.01	–	–

Compost data retrieved from Touceda-González et al. (2017).

The amendment with compost induced an immediate improvement of most fertility parameters of the barren soil. The resulting amended soil (compost:barren soil, 1:10, w/w) was slightly less acidic, richer in C and nutrients, and with higher CEC_e_ dominated by basic cations. The changes in metal concentrations induced by the incorporation of compost depended on the element and the fraction considered. The pseudo-total concentration of the metals analyzed did not significantly changed, except in the case of Zn and Pb which increased. Regarding available fractions, compost amendment significantly reduced the concentration of both, potentially available (EDTA-extractable) and readily available (NH_4_NO_3_ extractable) fractions of Al and Cu. The reduction of readily available Al and Cu (to 1/10 of the initial concentration) was particularly pronounced. Conversely, potentially and readily available fractions of Fe, Mn and Zn significantly increased after the incorporation of compost to the soil. Finally, the compost induced an increase of potentially available but not of ready available Pb concentration (Table 1).

### 3.2. Plant growth and elemental composition

The soil amendment with compost allowed the survival and growth of the planted poplar cuttings; although, an important number of dead or ill individuals was observed. Of the total number of plants 65% survived, and 42.5% were free of any pathological symptomatology or infestation. Most of the plant failures were observed in the non-inoculated treatments. Out of a total of 14 dead plants, 3 corresponded to P-NI, 4 to PT-NI and 3 were recorded in PT-MYC.

**Figure 1:**
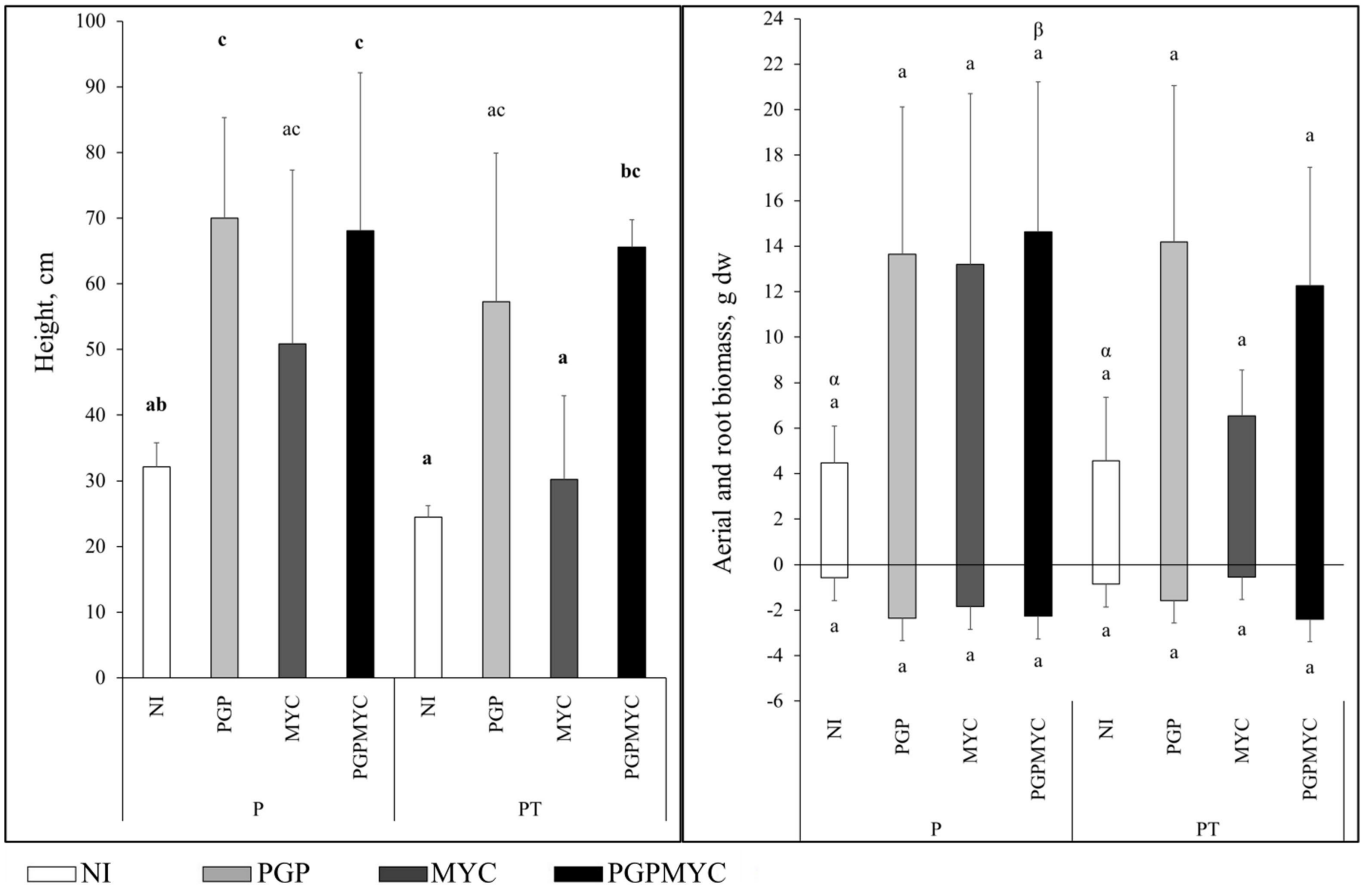
Growth in height and biomass of *Populus nigra* plants after 110 days. P, planted with *P. nigra* alone; PT, co-cropping of *P. nigra* and *Trifolium repens*; NI, not inoculated; PGP, inoculated with plant growth promoting bacteria; MYC, inoculated with mycorrhizae; PGPMYC, both inocula. Latin letters mark statistically significant difference (*p* < 0.05) between treatments. Greek letters mark differences in treatments that are close to statistical significance (*p* < 0.10).

The inoculation with PGP bacteria, applied separately or in combination with mycorrhizae, enhanced plant growth. The 2-ANOVA showed that the inoculum factor explained more variance of the variations in plant height and aerial biomass (*p* < 0.05, *η*^2^ > 29%) than the interaction inoculum-plant. Thus, irrespective of the monoculture or co-cropping pattern, the plants receiving PGP or PGPMYC were generally significantly higher than plants of other treatments; however, the effect of shoot biomass was less pronounced (only close to statistically significant values in the case of the shoot biomass of P-PGPMYC, *p* < 0.09; Figure 1).

**Figure 2:**
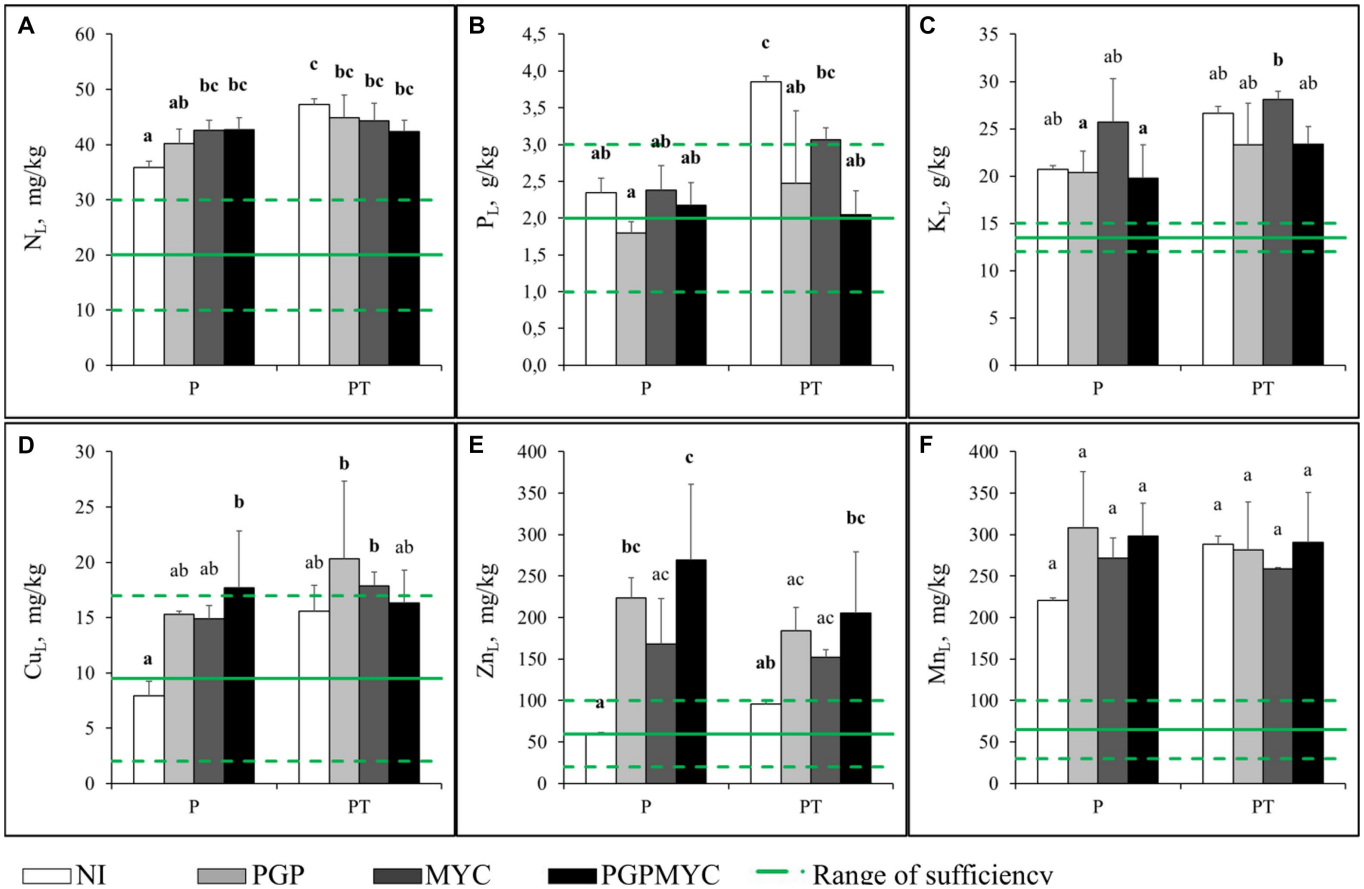
Different element concentrations in leaves of *Populus nigra* plants after 110 days of growth. Green broken lines represent the usual range of sufficiency in this kind of plant (Kabata-Pendias and Pendias, 2001; Baker and Pilbeam, 2007) and the continuous line is the arithmetic mean. **(A)** combustion analysis concentration of nitrogen (macronutrient), **(B)** acid digested concentration of phosphorous (macronutrient), **(C)** acid digested concentration of potassium (macronutrient), **(D)** acid digested concentration of copper (micronutrient and pollutant), **(E)** acid digested concentration of zinc (micronutrient and pollutant), **(F)** acid digested concentration of manganese (micronutrient and pollutant). P, planted with *Populus nigra* alone; PT, co-cropping of *P. nigra* and *Trifolium repens*; NI, not inoculated; PGP, inoculated with plant growth promoting bacteria; MYC, inoculated with mycorrhizae; PGPMYC, both inocula. Latin letters mark statistically significant difference (*p* < 0.05) between treatments.

In general, the cultivation pattern or inoculation did not significantly affect the plant ionome. Concentration of most TEs were below phytotoxicity levels (Cu: 15–30 mg/kg dw; Zn: 200–1,000 mg/kg dw; Mn: 500 mg/kg dw) found in literature (Kabata-Pendias and Pendias, 2001; Figure 2). Inoculation with PGP bacteria, alone or in combination with mycorrhizae, tended to increase trace element concentrations in leaves, especially when poplar was grown in monocropping, but the differences only were statistically significant for Cu and Zn in some treatments (Figures 2D–F).

**Figure 3:**
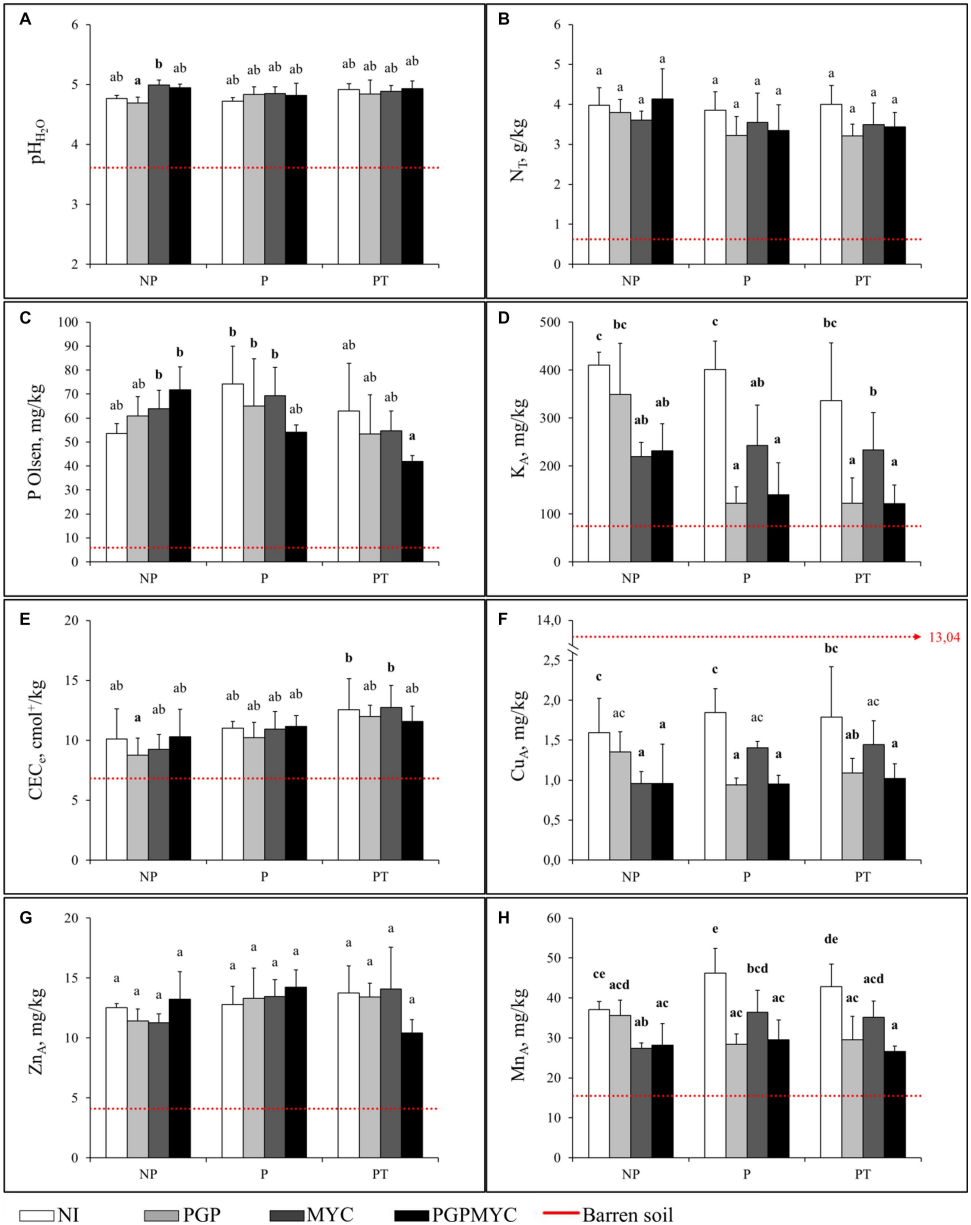
Main chemical fertility parameters and available concentrations of trace elements in the soil after 110 days of plant growth. Red dotted line is the barren soil reference level. **(A)** soil pH_H2O_ **(B)** total nitrogen, **(C)** available phosphorous, **(D)** available potassium, **(E)** effective cation exchange capacity, **(F)** available copper, **(G)** available zinc, **(H)** available manganese. NP, unplanted; P, planted with *Populus nigra* alone; PT, co-cropping of *P. nigra* and *Trifolium repens*; NI, not inoculated; PGP, inoculated with plant growth promoting bacteria; MYC, inoculated with mycorrhizae; PGPMYC, both inocula. Different Latin letters mark statistically significant difference (*p* < 0.05) between treatments.

In the absence of bioinocula or plants, an improvement of some physic-chemical properties of the compost-amended soil after 110 days in the glasshouse was observed. Thus, pH and P Olsen in non-planted amended substrate (NP-NI) were significantly higher than the initial values. A decrease of available Cu and an increase of readily available Mn were also observed at the end of the experiment. Inoculation with mycorrhizae, alone or in combination with PGP bacteria, induced additional increase of P Olsen and pH; conversely, the availability of Cu, Mn and K decreased in the soils receiving fungal bioinocula. The decrease of available K to values down to half of the concentration in non-inoculated soil (*p* < 0.05) and similar to those in non-amended soil, was especially pronounced (Figure 3).

At the end of the experiment the presence of plants induced some changes in the physic-chemical characteristics of the substrate. 2-ANOVA analysis showed that plant factor was significant in explaining the variance of P Olsen and CEC_e_, although, the effect was low (*η*^2^ < 29%, both). The most important difference between the non-planted and planted substrate was a decrease of the K availability when plants inoculated with PGP of PGPMYC were present. A similar behavior was observed for Cu an Mn in the case of PGP inoculation treatment. Moreover, P Olsen tended to be lower in pots with plants receiving PGPMYC inocula than in non-planted pots. The 2-ANOVA analyses showed that the inoculation explained a significant amount of the variance of the available Cu, Mn, and K concentrations (*p* < 0.05, *η*^2^ > 42%, all), while the interaction between the inoculum and the plant factors contributed to explain variations of the P Olsen content (*p* < 0.05, *η*^2^ < 17%). Finally, CEC_e_ tended to increase in planted pots, especially in the case of the co-cropping, and the lower values of total N were observed in pots with bioinoculated plants (Figure 3).

As observed in non-planted pots and irrespective of the bioinocula or cropping treatment, pH increased during the experiment (Figure 3).

### 3.3. Functional diversity of the microbial community

The amendment of the barren soil with compost dramatically improved the bacterial C substrates degradation activity (A, expressed as average well color development, AWCD) and richness (S) (barren soil A = 0.08 ± 0.02; barren soil S = 6.0 ± 1.2) in all the groups in relation to the barren soil (Table 2). Moreover, the inoculation with mycorrhiza slightly increased degradation activity. According to 2-ANOVA analyses, inoculum factor contributed to explain the variance of A and S (*p* < 0.05, *η*^2^ > 16%, both). The factor plant (*p* < 0.05, *η*^2^ = 17%) and the interaction inoculum-plant (*p* < 0.05, *η*^2^ = 21%) were also significant for the variable A. Margalef richness (M) and McIntosh dominance (I) were mostly influenced by factor plant (*p* < 0.05, *η*^2^ > 22%, both). Interaction inoculum-plant was also explanatory for I (*p* < 0.05, *η*^2^ = 24%). As for the Shannon-Wiener diversity (H) the sole explanatory factor was inoculum (*p* < 0.05, *η*^2^ = 14%). Considering treatments separately, 1-ANOVA indicated that the effect of inoculation on soil bacterial functional diversity depended on the cropping pattern (Table 2). In the absence of plant (NP), the effect of the bioinoculation was not statistically significant. In planted pots the highest A and S were detected in the treatments using PGP alone or in combination with mycorrhiza. In comparison with the monocropped non-inoculated reference (P-NI), the increase in activity was statistically significant in the case of P-PGP while richness was statistically higher in PT-PGPMYC. Coherent, although not statistically significant increases on H index were also observed in planted pots inoculated with PGP or PGPMYC. Additional differences, attributable to the interaction inoculum-plant, were observed when analyzing both plantation and inoculation patterns at the same time. Thus, in NP-MYC treatment A was significantly higher and I significantly lower than in P-MYC and PT-MYC treatments. Index I was lower in NP-PGPMYC than in P-PGPMYC (Table 2).

**Table 2:** Main microbiological parameters of activity, richness and diversity with means and standard deviation.

Treatments	A		S		M		H		I	
	Mean	±S.D.	Mean	±S.D.	Mean	±S.D.	Mean	±S.D.	Mean	±S.D.
NP	NI	0.97 cd	0.36	26.0 ab	0.7	17.4 ab	2.1	4.44 a	0.09	0.96 ab	0.04
	PGP	0.77 ad	0.24	25.2 ab	2.7	17.9 ab	0.3	4.49 a	0.32	0.97 ac	0.01
	MYC	1.26 d	0.46	26.6 ab	3.0	16.7 ab	0.7	4.39 a	0.26	0.92 a	0.01
	PGPMYC	1.44 d	0.62	27.4 ab	5.0	16.5 a	1.5	4.40 a	0.41	0.92 ab	0.02
P	NI	0.36 ab	0.08	20.3 a	1.7	18.8 ab	1.8	4.17 a	0.31	1.03 bc	0.05
	PGP	1.31 cd	0.51	28.3 ab	3.6	18.5 ab	2.8	4.61 a	0.15	0.97 ac	0.06
	MYC	0.32 a	0.09	21.0 ab	4.6	20.8 b	2.0	4.11 a	0.28	1.08 c	0.06
	PGPMYC	0.67 ad	0.49	25.8 ab	4.5	20.6 ab	3.4	4.48 a	0.25	1.09 c	0.08
PT	NI	0.50 ac	0.19	24.3 ab	4.4	20.0 ab	1.1	4.29 a	0.24	1.03 bc	0.05
	PGP	1.20 cd	0.02	28.7 ab	0.6	17.6 ab	0.3	4.58 a	0.04	0.94 ab	0.01
	MYC	0.55 ac	0.45	23.0 ab	3.7	19.7 ab	1.5	4.29 a	0.22	1.06 c	0.08
	PGPMYC	1.27 cd	0.31	28.6 b	1.5	17.5 ab	4.6	4.58 a	0.08	0.94 ab	0.02

Statistical significant differences between treatments (*p* < 0.05) marked with Latin letters. NP, unplanted; P, planted with *Populus nigra*; PT, planted with *Populus nigra* and *Trifolium repens*; NI, not inoculated; PGP, inoculated with plant growth promoting bacteria; MYC, inoculated with mycorhiza; PGPMYC, inoculated with plant growth promoting bacteria and mycorhiza; A, average activity, expresed as the average well color development (AWCD); S, total number of metabolic species; M, Margalef index of effective richness of species; H, Shannon-Wiener index of diversity; I, McIntosh index of dominance.

**Figure 4:**
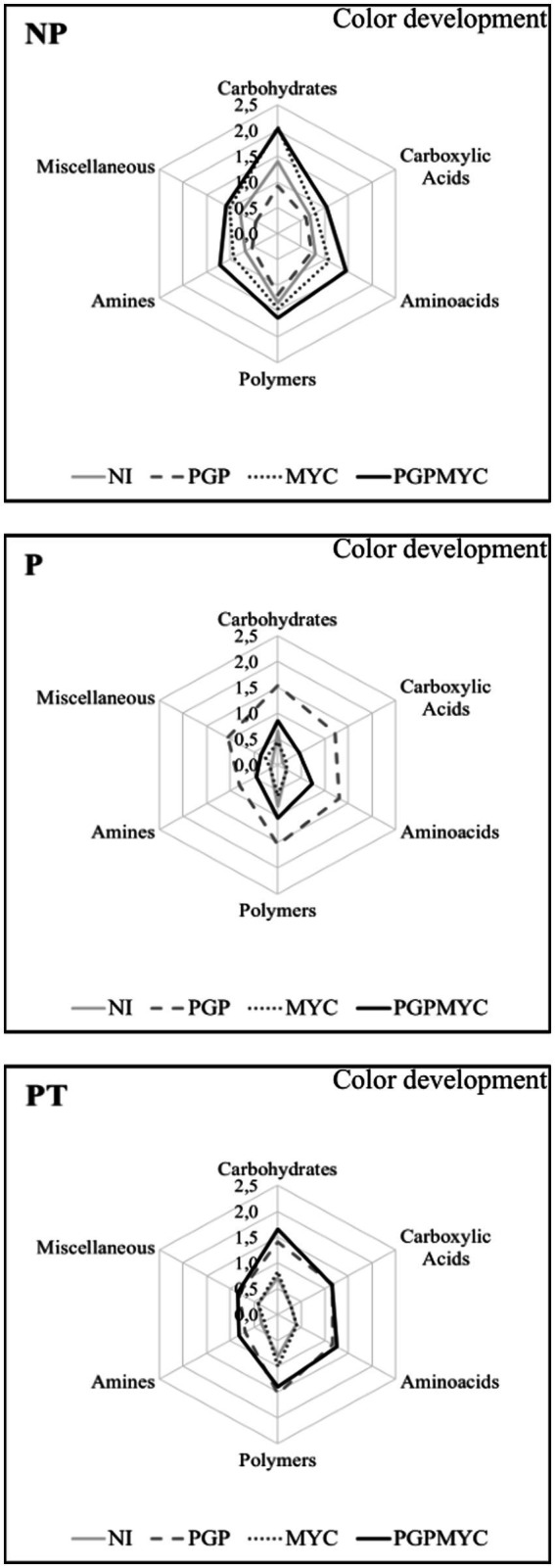
Differences in microbial communities according to preferential use (color development) of the different types of substrates. NP, unplanted; P, planted with *Populus nigra* alone; PT, co-cropping of *P. nigra* and *Trifolium repens*; NI, not inoculated; PGP, inoculated with plant growth promoting bacteria; MYC, inoculated with mycorrhizae; PGPMYC, both inocula.

The microbial communities present in pots receiving different treatments showed preferential use of certain substrates. In general, the bacterial communities showed a good degradation activity of carbohydrates and polymers, however, in non-planted soils the use of amines was relatively higher while use of carboxylic acid was relatively lower than in planted substrates inoculated with PGP or PGPMYC. Besides, a lower general activity, P-PGPMYC differs from P-PGP in the relatively low use of carboxylic acids, a feature shared with unplanted (NP) and not inoculated with bacteria (NI and MYC; Figure 4).

**Figure 5:**
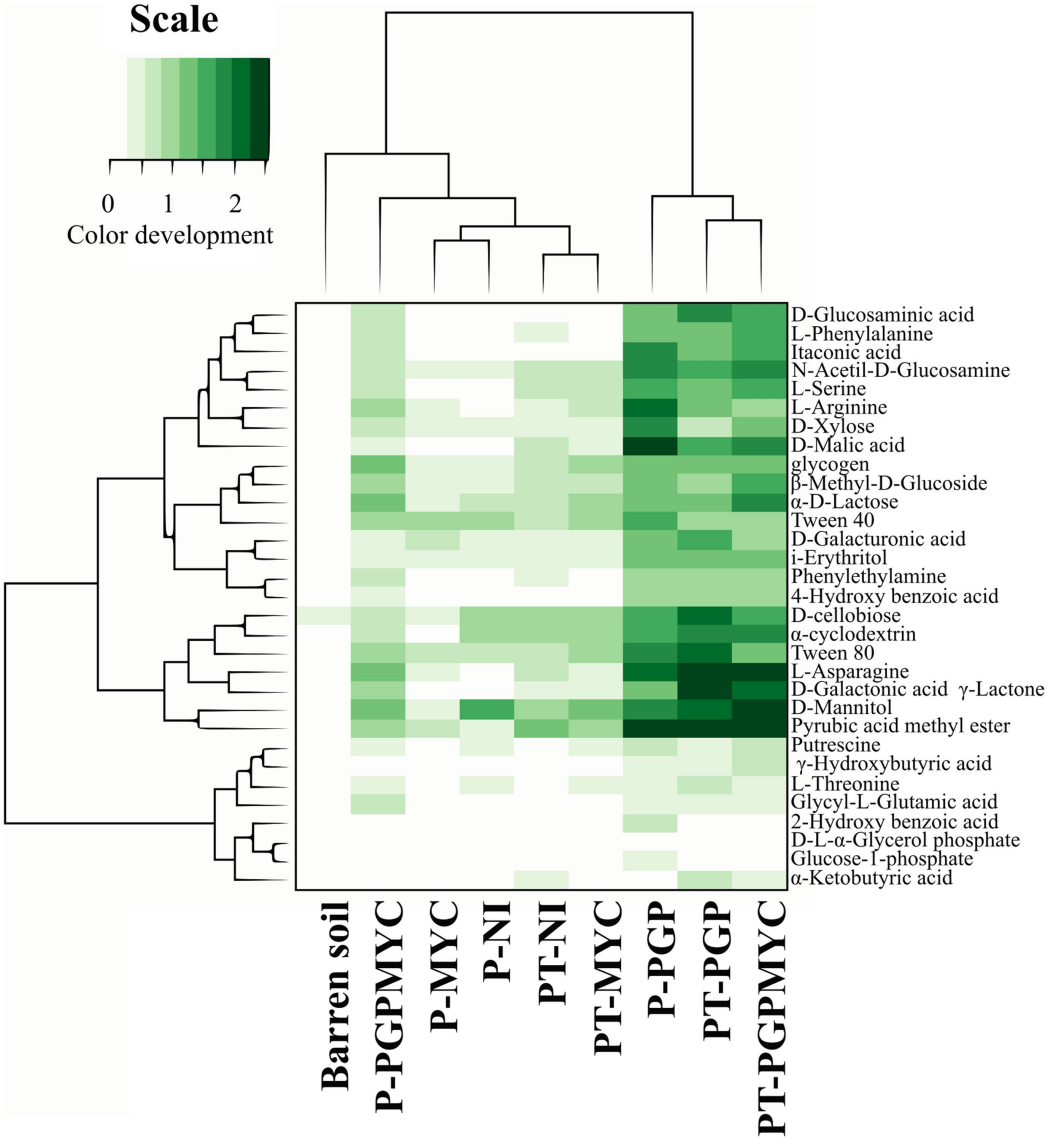
Dendrogram grouping microbial communities of the planted treatments according to preferential use (color development) of individual substrates. Simultaneously, the substrates are grouped according to the general usage. P, planted with *Populus nigra* alone; PT, co-cropping of *P. nigra* and *Trifolium repens*; NI, not inoculated; PGP, inoculated with plant growth promoting bacteria; MYC, inoculated with mycorrhizae; PGPMYC, both inocula.

The analysis of the use of individual substrates allowed the detection of differences between microbial communities in planted pots receiving different bioinocula. The dendogram obtained from an initial exploratory statistical analysis including all samples was strongly influenced by the high distances found among NP samples; therefore, the clustering obtained did not offer relevant information (data not shown). In Figure 5, the dendrogram grouping planted samples according to the bacterial degradation patterns showed two clusters, one including most of the samples inoculated with PGP or PGPMYC and a second cluster grouping samples non-inoculated or inoculated with mycorrhiza. The algorithm also arranged substrates by degree of usage. All communities showed a low use of the carboxylic acids 2-hydroxybenzoic and alpha-ketobutyric and of the phosphate containing compounds glucose-1-phosphate and D-L-alpha-glycerol-phosphate. The bacterial degradation on the aminoacids L-threonine, glycyl-L-glutamic acid and phenylalanine of the two amines tested (putrescine and phenylethylamine) and of several other carboxylic acids (gamma-hydroxybutyric, itaconic, glucosaminic and 4-hydroxybenzoic) was also low in planted pots non-inoculated or inoculated with mycorrhiza. The communities of the co-cropping treatments PT-NI and PT-MYC degraded serine, malic acid and mannitol more efficiently than those of the monocrop treatment P-NI or P-MYC (Figure 5).

## 4. Discussion

The compost amendment applied to the mine soil tackled most of the issues this barren soil by increasing the pH, providing C and nutrients, enhancing CEC_e_ and reducing the availability of some trace elements such as Cu. Several studies have demonstrated that soil amendments can facilitate revegetation of contaminated soils by providing fertility in the form of nutrients and highly humified organic matter which increases the number of cation exchange sites, and overall decreases mobility of toxic elements through chemical precipitation, metal binding to the colloids and formation of stable organometallic complexes, neutralizes the pH and improves structure (Soler-Rovira et al., 2010; Park et al., 2011; Becerra-Castro et al., 2018). Compost was also successfully used in field phytostabilization trials. For instance, Touceda-González et al. (2017) applying the same compost than in this study as soil amendment in a field trial obtained a pronounced significant decrease of bioavailable (NaNO_3_ extractable) Cu concentration from 33 mg/kg to 1.2 mg/kg after 3 years. These authors highlighted the need for longer-term trials to evaluate the effect of organic matter mineralization on Cu mobility.

The observed strong decrease of Cu and Al availability in the amended mine soil can be attributed to both the increase of about 1 unit in pH and the incorporation of organic compounds of the compost. It is well known that the availability of TEs decreases as pH tends to neutrality (Kabata-Pendias and Pendias, 2001). Humic acids of the compost can reduce Cu availability by forming stable complexes, but the increase in pH was more important in the immobilization of this element in a field study (Soler-Rovira et al., 2010). Conversely the compost incorporation to the mine soil increased the availability of Zn and, to a lesser extent, of Mn. Other authors have found increased availability in Zn and Mn (but not Cu) after the application of organic amendments and have attributed it to the formation of element-specific soluble organo-metal compounds (Maqueda et al., 2015). On the other hand, the incorporation of available Mn and especially Zn to the soil with the amendment cannot be discarded taking into account that the total concentration of certain TEs in the compost used in the experiment was similar (Mn and Cu) or higher (Zn) than in the mine soil. The increase in the availability of Mn after the plant growth period can be explained by processes related with organic matter dynamics causing a steady release of this element from immobile pools. Other authors pointed that in contaminated soils amended with organic substrates a delayed increase of available pollutants through changes in the organic matter and physic-chemical properties of the soil can take place (Clemente et al., 2015). In turn, the decrease of Cu availability detected at the end of the experiment agrees with the observed increase in pH.

The improvement of soil fertility conditions due to the amendment allowed the plant growth. Despite, the observed death and disease rates indicated a certain level of stress for the plants. The fact that in co-cropping plant performance was slightly worse, could mean that *Trifolium repens* competed with *Populus nigra*; however, the co-cropping improved N and P concentrations in poplar leaves. The co-cropping did not induce important changes on soil physic-chemical properties and TEs availability, chich allows to discard a deleterious effect of the cultivation pattern through increasing soil toxicity. The incorporation of legumes in phytostabilization cropping patterns is a technique that seeks to tackle with nutrient deficiencies of degraded soils. In a field study, perennial grass mixtures that included the legume *Anthyllis vulneraria* achieved higher cover, biomass and improved nutritional content in a trace element polluted soil compared to a grass-only control (Frérot et al., 2006). Intercropping with leguminous plants can improve nutrient availability through N_2_ fixation, fixed N transfer and P mobilization due to rhizosphere acidification (Kidd et al., 2015). In the present study, nonetheless, nutrient concentration in leaves were not related to an improved plant growth. Lachapelle et al. (2020) who studied a co-cropping system with *Trifolium* sp. and the woody species *Salix interior* attributed the lack of a positive effect of the co-cropping pattern to resource competition. The same authors found better phytoextraction parameters in co-cropping comparing with monoculture. In our study the Cu, Zn and Mn phytoextracted by poplar was similar in the monoculture and the co-cropping systems.

The bioinocula applied, especially PGP and PGPMYC treatments, induced a generalized increase in plant survival and development. These improvements are attributable to the PGP properties of the bacterial mixture used, including rhizobacteria able to produce IAA, biosurfactants and siderophores, and to solubilize phosphate. The bacterial production of phytohormone IAA may have had a direct effect on plant growth; on the other hand, the observed increase of P Olsen in NP pots with all bioinocula, especially with PGPMYC, confirms the potential of the microorganisms used for increasing nutrient availability for plants. The decrease of available K, Cu and Mn, and of P Olsen in planted inoculated soils is likely related to the plant uptake of these elements. The more pronounced reduction observed in treatments with better plant development (PGP and PGPMYC) supports this explanation.

The presence of mycorrhizae can stimulate plant growth provided that potential benefits offset the energy cost of maintaining the symbiotic relationship (Marschner, 2011). In this experiment, the effect of mycorrhiza inoculation on plant growth is negligible. So, either the cost of maintaining the relationship equaled the benefit or the symbiosis was not established. Considering that the mycorrhization was performed with commercial inoculum, it is likely that these organisms are not adapted to the type of soil and/or plants used in this experiment (Madejón et al., 2021). Alternatively, mycorrhizal inoculation might have funneled phytotoxic soil elements to the plant, as noted by Phanthavongsa et al. (2017), forcing plants to invest resources in maintaining homeostasis. Mycorrhizae could have also infected *T. repens* increasing resource competition with *P. nigra*, which may explain the poorer growth of the later in PT-MYC than in P-MYC. According to Huang et al. (2014), infection rate is more determinant than inoculum size in the short-term effects of inoculation with arbuscular mycorrhizae.

No clear synergistic effect of PGP bacteria and mycorrhizae was observed; globally, it seems that the effect of the bacteria masked any potential plant growth promotion induced by mycorrhizae. However, as inoculation with mycorrhizae alone was rather neutral, the benefial effects of the combined inocula were likely due to the PGP bacterial strains. Furthermore, different combination of PGPR and mycorrhizae may have different effects in different environments or on different plants (Santoyo et al., 2021). Therefore, further research is needed to explore other bacterial-mycorrhizal combinations adequate for improving phytostabilization techniques.

Although the aim of inoculation, and mycorrhization in particular, was promoting plant growth, it is worth pointing out the importance of the results obtained for the inoculated non-planted soil, which give information on the modifications of soil properties and nutrient availability induced by the bioinocula applied.

Soil amendment drastically improved the average bacterial degradation capacity of C sources as well as functional diversity, likely due to the incorporation of the microbiota present in the compost and the general improvement of physic-chemical properties and the abundance and diversity of C compounds of the amended soil. Differences in activity, richness of metabolic species, dominance and preferential use of substrate in unplanted (NP) and planted (P and PT) pots indicate that plant presence is an important factor shaping the soil microbiological community. In NP a very active community developed, whereas in P and PT a less active but richer community was detected, which might be attributed to a rhizosphere effect observed before by numerous authors (Söderberg et al., 2004; Selmants et al., 2005).

In the absence of inoculum or with mycorrhiza, the activity and diversity of the soil community in the co-cropping pattern tended to be higher than in monoculture. In other studies, the development of mixed plant covers also increased soil microbial diversity when a second plant species intervenes (Benizri and Amiaud, 2005). However, when the bacterial inocula was present (PGP and PGPMYC), the differences between mono and co-cropping tended to disappear or be less consistent. These treatments with PGP bacteria significantly improved the poplar survival and growth, which probably masked the influence of the co-cropping on the bacterial activity.

In general, bacterial activity and diversity were higher in the treatments with better plant development, which suggest that the positive feedback between plants and rhizosphere microorganisms is particularly important in soils of low fertility and problems of acidity and high availability of toxic TEs. In these problematic soils the C supply in form of plant exudates is probably particularly important for the microbial proliferation (Alawiye and Babalola, 2019); in turn, the recruitment of beneficial microbial community in the rhizosphere is essential for the plant development in such stressing environments. Low activity in P-NI, P-MYC, PT-NI and PT-MYC is in agreement with plant mortality and reduced plant growth.

## 5. Conclusion

The incorporation of a soil amendment rich in organic matter and nutrients to a very poor acid mine soil was essential for improving nutrient status, correct acidity and decrease Al and Cu availability, which allowed the survival and growth of poplar trees (*Populus nigra* L.) cultivated in monocropping or co-cropping with clover (*Trifolium repens* L.) in the amended soil.

The survival and development of poplar was significantly improved with bioinocula containing a consortium of bacterial strains with plant promoting properties (PGP) isolated from substrates contaminated with trace elements; however, the application of commercial mycorrhizal inoculum did not have a significant effect. The growth of the poplar trees also did not improve with the co-cropping with clover. The increase in P availability and the reduction of Cu availability seems to be among the mechanisms of plant growth promotion of the bacterial consortium.

The soil amendment with a C and nutrient rich substrate dramatically increased soil bacterial activity and functional diversity; however, biological properties were particularly improved when the poplar performance was stimulated by the application of PGP bacterial inocula. These results suggest that the positive feedback between plants and rhizosphere microorganisms is probably particularly important in soils of low fertility and problems of acidity and high availability of toxic TEs. In future works it will be interesting to verify the persistence of the inoculated organisms and to test the performance of native mycorrhizae directly isolated from the surrounding of the studying area.

In sum, this work provides evidence on the benefits of using simple and inexpensive soil amendments in combination with tolerant plants and effective bioinoculants for the management of polluted sites.

## Data availability statement

The original contributions presented in the study are included in the article, further inquiries can be directed to the corresponding author.

## Author contributions

CM and PK are responsible for trial design, supervision and discussion of results. MR-E contributed to all steps of the work presented. CM and AP-F were involved in the discussion, writing and revision of the manuscript. JR-C, YS and BR-G participated in the setup of the experiments and analytical work. All authors approved the submitted version.

## Funding

This work was funded by European Commission-V Interreg Sudoe Program (Phy2SUDOE project - SOE4/P5/E1021), and the Xunta de Galicia (GRCED431C 2022/40), co-funded by FEDER (EU).

## Conflict of interest

The authors declare that the research was conducted in the absence of any commercial or financial relationships that could be construed as a potential conflict of interest.

## Publisher’s note

All claims expressed in this article are solely those of the authors and do not necessarily represent those of their affiliated organizations, or those of the publisher, the editors and the reviewers. Any product that may be evaluated in this article, or claim that may be made by its manufacturer, is not guaranteed or endorsed by the publisher.
